# Supplementary material for: Classification of likely functional class for ligand binding sites identified from fragment screening
Source: Commun Biol. 2024 Mar 13;7:320. doi: 10.1038/s42003-024-05970-8 (PMC10937669; doi:10.1038/s42003-024-05970-8)
Supplement: Supplementary file 2 — Supplementary Information [file 42003_2024_5970_MOESM2_ESM.pdf]

Classification of likely functional class for ligand binding sites identified from fragment screening

Supplementary Information

Javier S. Utgés, Stuart A. MacGowan, Callum M. Ives[1]

and Geoffrey J. Barton*

Division of Computational Biology, School of Life Sciences,

University of Dundee, Scotland, UK

*Correspondence to: gjbarton@dundee.ac.uk

[1.] Present address: Department of Chemistry and Hamilton Institute, Maynooth University, Maynooth, Ireland. callum.ives@mu.ie

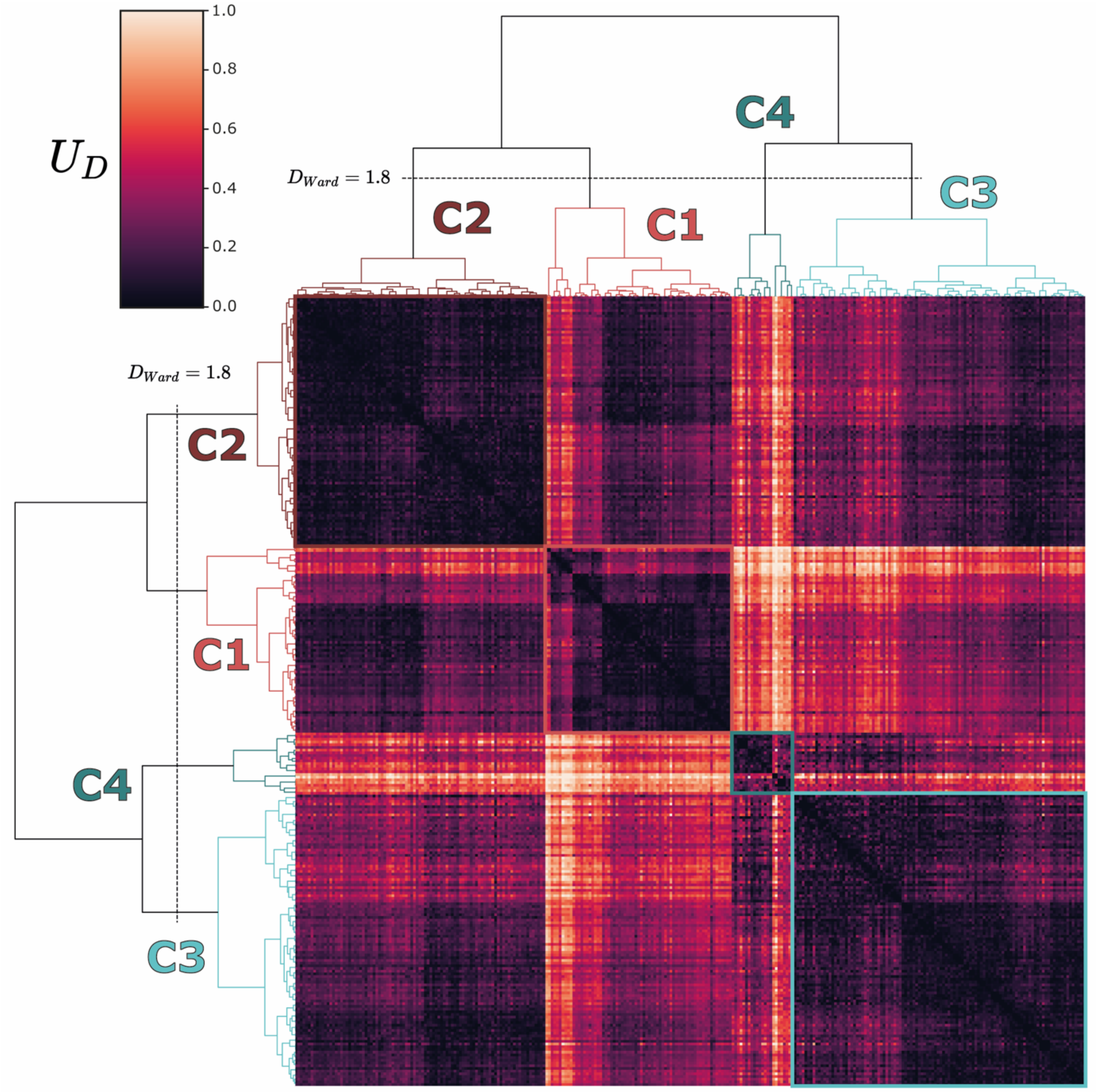

Supplementary Figure 1. Heat map of the U distance, U_D, matrix of the 293 defined binding sites clustered by the Ward hierarchical clustering method [1] implemented in SciPy [2]. The tree is cut at D_{Ward} = 1.8, giving four clear clusters. These clusters are labelled so they correspond to the ones obtained with K-means [3]. Clusters in the heatmap are represented by dark squares around the diagonal. U_D is a distance; therefore, clusters include sites that are similar to each other, and present lower distances (dark colour).

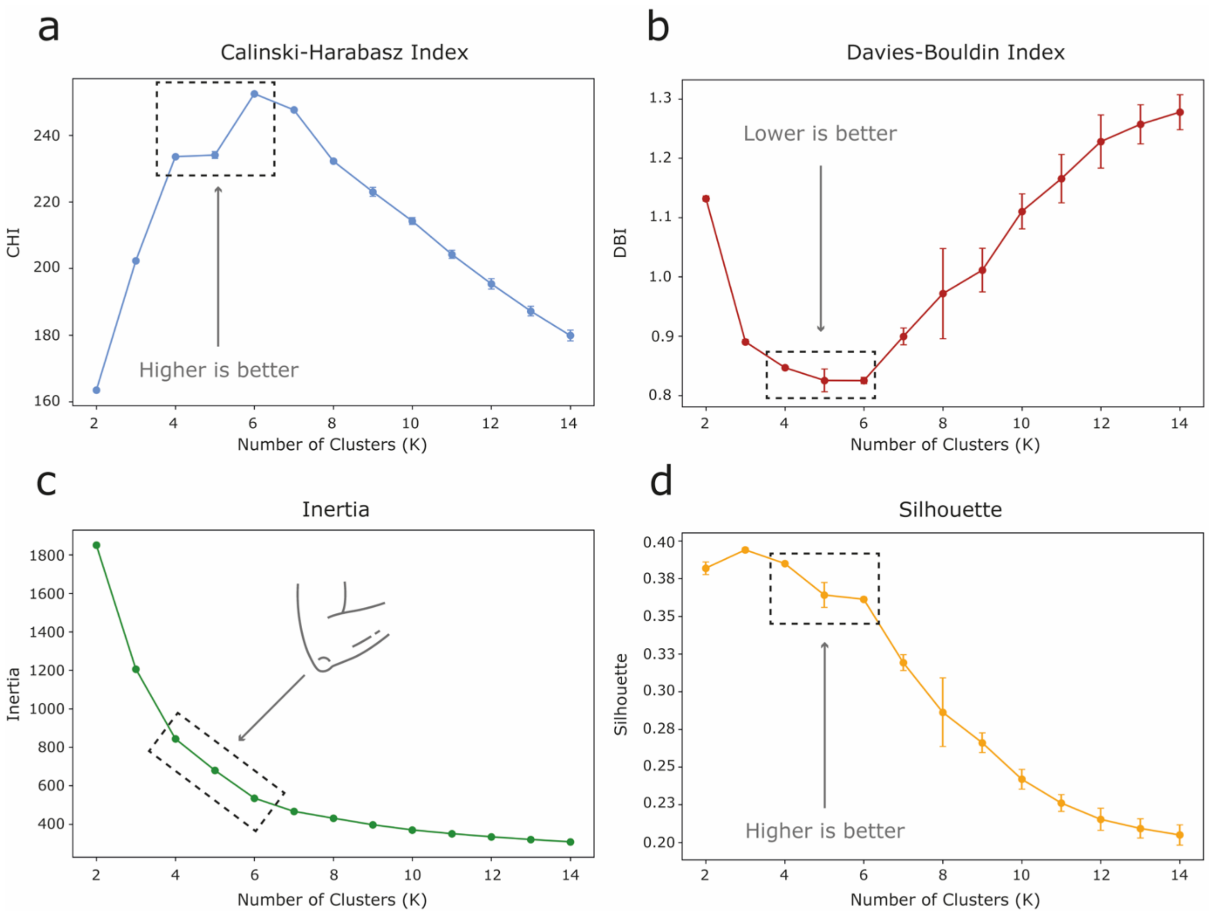

Supplementary Figure 2. Cluster analysis to assess the quality of the K-means clustering. For each $K \in [2, 14]$, clustering is bootstrapped 1,000 times with different initial random states. Error bars indicate 1 SD. (A) Calinski-Harabasz Index (CHI) [4]; (B) Davies-Bouldin Index (DBI) [5]; (C) Inertia [6]; (D) Silhouette [7]. All methods agree the optimal clustering of this dataset lies in $K \in [4, 6]$.

Supplementary Note 1: MLP ablation studies

A thorough hyperparameter optimisation was carried out by examining the effect that a series of hyperparameter changes have on the prediction accuracy relative to our current ML setup, labelled as **current**. Sixty-four single-hyperparameter changes were performed, one at a time. For each variation, 100 models were trained with different seeds and the average validation accuracies compared to our current multilayer perceptron (MLP). Sixty-four pairwise t-tests were conducted to compare the accuracy means, and Benjamini-Hochberg correction [8] applied. FDR and $\Delta_{\mathrm{acc}} = \mathrm{acc}_{\mathrm{VARIANT}} - \mathrm{acc}_{\mathrm{CURRENT}}$ are used to describe the results, where $\mathrm{acc}_{\mathrm{CURRENT}}$ is the average validation accuracy of our current ML setup across the 100 seeds, and $\mathrm{acc}_{\mathrm{VARIANT}}$ is the average accuracy across 100 seeds of each one of the 64 variant models.

$\Delta_{\mathrm{acc}} < 0$ will represent a decrease in performance respect our current ML architecture, whereas $\Delta_{\mathrm{acc}} > 0$ will mean a higher accuracy.

The results of these analyses are described below and graphically represented in Supplementary Figure 3 and Supplementary Table 1.

Removing the single hidden layer resulted in a significant decrease in accuracy, $\Delta_{\mathrm{acc}} = -11\%$ ($\mathrm{FDR} < 0.05$).

The addition of more layers did not improve accuracy: 2-layer $\Delta_{\mathrm{acc}} = -1\%$ ($\mathrm{FDR} < 0.05$), 10-layer $\Delta_{\mathrm{acc}} = -8.9\%$ ($\mathrm{FDR} < 0.05$), or was not statistically different from our current setup baseline: 5-layer $\Delta_{\mathrm{acc}} = -0.15\%$ ($\mathrm{FDR} = 0.42$).

The addition of neurons $\mathrm{N}_{\mathrm{neurons}} = [11, 20, 25, 50, 100]$ in the single layer did not improve the current accuracy ($\mathrm{FDR} > 0.05$).

The removal of neurons did not have an effect of performance $\mathrm{N}_{\mathrm{neurons}} = [4, 5, 6, 7, 8, 9]$ ($\mathrm{FDR} > 0.05$), or a significant negative effect for 1 neuron, $\Delta_{\mathrm{acc}} = -15\%$ ($\mathrm{FDR} < 0.05$), 2 neurons $\Delta_{\mathrm{acc}} = -4\%$ ($\mathrm{FDR} < 0.05$), and 3 neurons, $\Delta_{\mathrm{acc}} = -1\%$ ($\mathrm{FDR} < 0.05$).

This result suggests that 5 neurons on a single hidden layer might be enough to achieve a comparable accuracy to our current model.

The usage of different activation functions either negatively affected the accuracy of the MLP ($\Delta_{acc} < 0$) or had no effect ($FDR > 0.05$).

Most weight initialisers were tested and either negatively affected the accuracy of the MLP ($\Delta_{acc} < 0$) or had no effect ($FDR > 0.05$). However, RandomNormal, RandomUniform, and TruncatedNormal did improve the accuracy but by less than 1%, $\Delta_{acc} < +1\%, (FDR < 0.05)$.

Regarding dropout rates, a rate = 75%, negatively affected prediction $\Delta_{acc} < -2\%, (FDR < 0.05)$. Lower dropout rates: 0.1, 0.25, and 0.33 did improve the accuracy, but the effect size is very small, $\Delta_{acc} < +1\%, (FDR < 0.05)$.

This result agrees with the effect of the removal of neurons per layer and shows that fewer neurons on a single hidden layer might be enough to achieve a comparable accuracy to our current model, as dropping them out has no effect.

Different loss functions resulted in terrible loss of accuracy $\Delta_{acc} \approx -50\%, (FDR < 0.05)$. This is expected as they are not appropriate for a multi-label classifier, unlike sparse categorical cross entropy.

Regarding optimisers, they either severely negatively affected accuracy $\Delta_{acc} \approx -30\%, (FDR < 0.05)$, had no significant effect ($FDR > 0.05$), or very slightly improved accuracy, such as RMSProp $\Delta_{acc} < +1\%, (FDR < 0.05)$.

Extreme learning rates of 0.001 (too small), and 1.0 (too big) negatively affected prediction $\Delta_{acc} < -5\%, (FDR < 0.05)$. Intermediate rates had either no significant effect ($FDR < 0.05$) nor relevant $|\Delta_{acc}| < 1\%$.

Overall, implementing kernel, bias, or activity regularisation techniques did not improve prediction accuracy, but worsened it $\Delta_{acc} \in [-2.56, -0.46], (FDR < 0.05)$.

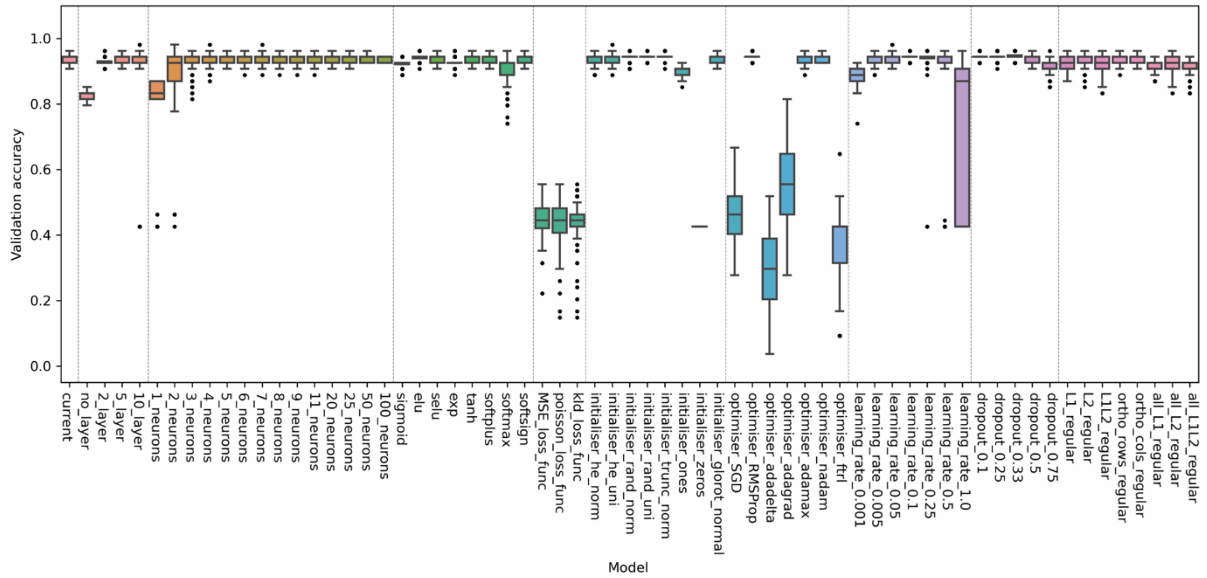

Supplementary Figure 3. Ablation study performed on the MLP. Sixty-four single hyperparameter changes are conducted one at a time to explore the hyperparameter space and the effect they have on the prediction accuracy relative to our current ML setup, labelled as *current*. Box and whiskers represent the distribution of validation accuracies across 100 random seeds. Dashed lines mark the separation between different hyperparameters: number of layers, neurons, activation, loss functions, weight initialisers, optimisers, learning, dropout rates, and regularisation techniques.

Model	Validation accuracy	Δ_{acc}	FDR
CURRENT	**0.94**	**-**	**-**
**no_layer**	**0.83**	**-11.00**	**0.00**
**2_layer**	**0.93**	**-1.00**	**0.00**
5_layer	0.94	-0.15	0.42
**10_layer**	**0.85**	**-8.93**	**0.00**
**1_neurons**	**0.79**	**-14.93**	**0.00**
**2_neurons**	**0.90**	**-4.15**	**0.00**
**3_neurons**	**0.93**	**-1.06**	**0.00**
4_neurons	0.94	-0.28	0.24
5_neurons	0.94	-0.13	0.54
6_neurons	0.94	-0.24	0.26
7_neurons	0.94	-0.39	0.08
8_neurons	0.94	-0.39	0.08
9_neurons	0.94	-0.15	0.49
11_neurons	0.94	0.09	0.65
20_neurons	0.94	0.07	0.68
25_neurons	0.94	-0.04	0.83
50_neurons	0.94	-0.02	0.91
100_neurons	0.94	-0.11	0.49
**sigmoid**	**0.92**	**-1.52**	**0.00**
elu	0.94	0.15	0.38
selu	0.94	-0.26	0.15
**exp**	**0.93**	**-1.17**	**0.00**
tanh	0.94	-0.15	0.41
softplus	0.93	-0.69	0.00
**softmax**	**0.90**	**-3.98**	**0.00**
softsign	0.94	-0.41	0.02
**MSE_loss_func**	**0.44**	**-49.81**	**0.00**
**poisson_loss_func**	**0.44**	**-50.31**	**0.00**
**kld_loss_func**	**0.44**	**-50.00**	**0.00**
initialiser_he_norm	0.94	-0.20	0.37

initialiser_he_uni	0.94	-0.26	0.21
initialiser_rand_norm	0.94	0.41	0.02
initialiser_rand_uni	0.94	0.44	0.01
initialiser_trunc_norm	0.95	0.59	0.00
**initialiser_ones**	**0.90**	**-3.96**	**0.00**
**initialiser_zeros**	0.43	**-51.35**	**0.00**
initialiser_glorot_normal	0.94	-0.06	0.79
**optimiser_SGD**	0.47	**-47.26**	**0.00**
optimiser_RMSProp	0.95	0.54	0.00
**optimiser_adadelta**	0.29	**-64.50**	**0.00**
**optimiser_adagrad**	0.55	**-38.83**	**0.00**
optimiser_adamax	0.94	0.11	0.60
optimiser_nadam	0.94	0.13	0.48
**optimiser_ftrl**	0.35	**-59.24**	**0.00**
**learning_rate_0.001**	**0.89**	**-4.98**	**0.00**
learning_rate_0.005	0.94	-0.44	0.04
learning_rate_0.05	0.94	-0.33	0.06
learning_rate_0.1	0.94	0.31	0.05
learning_rate_0.25	0.93	-0.83	0.27
**learning_rate_0.5**	**0.88**	**-5.70**	**0.00**
**learning_rate_1.0**	0.70	**-24.37**	**0.00**
dropout_0.1	0.95	0.56	0.00
dropout_0.25	0.95	0.61	0.00
dropout_0.33	0.95	0.80	0.00
dropout_0.5	0.94	0.11	0.54
**dropout_0.75**	**0.92**	**-2.02**	**0.00**
**L1_regular**	**0.93**	**-1.48**	**0.00**
L2_regular	0.94	-0.46	0.04
**L1L2_regular**	**0.92**	**-1.93**	**0.00**
ortho_rows_regular	0.94	-0.46	0.02
ortho_cols_regular	0.94	-0.09	0.62
**all_L1_regular**	**0.92**	**-2.11**	**0.00**

**all_L2_regular**	**0.92**	**-1.70**	**0.00**
**all_L1L2_regular**	**0.91**	**-2.56**	**0.00**

Supplementary Table 1. Ablation study performed on the MLP. Sixty-four single hyperparameter changes are conducted one at a time to explore the hyperparameter space and the effect they have on the prediction accuracy relative to our current ML setup, labelled as *CURRENT*. Validation accuracy represents the validation accuracy average across 100 random seeds. Δ_{acc} represents the difference in performance between the variant MLP model and our current setup. Negative values result from a decrease in performance, whereas positive ones mean an improvement in classification accuracy. FDR was employed to assess the significance of these differences. Rows are coloured in green when $\Delta_{acc} > 0$, orange when $-5 < \Delta_{acc} < 0$, and red if $\Delta_{acc} \leq -5$. Rows where $|\Delta_{acc}| \geq 1$ are in bold font.

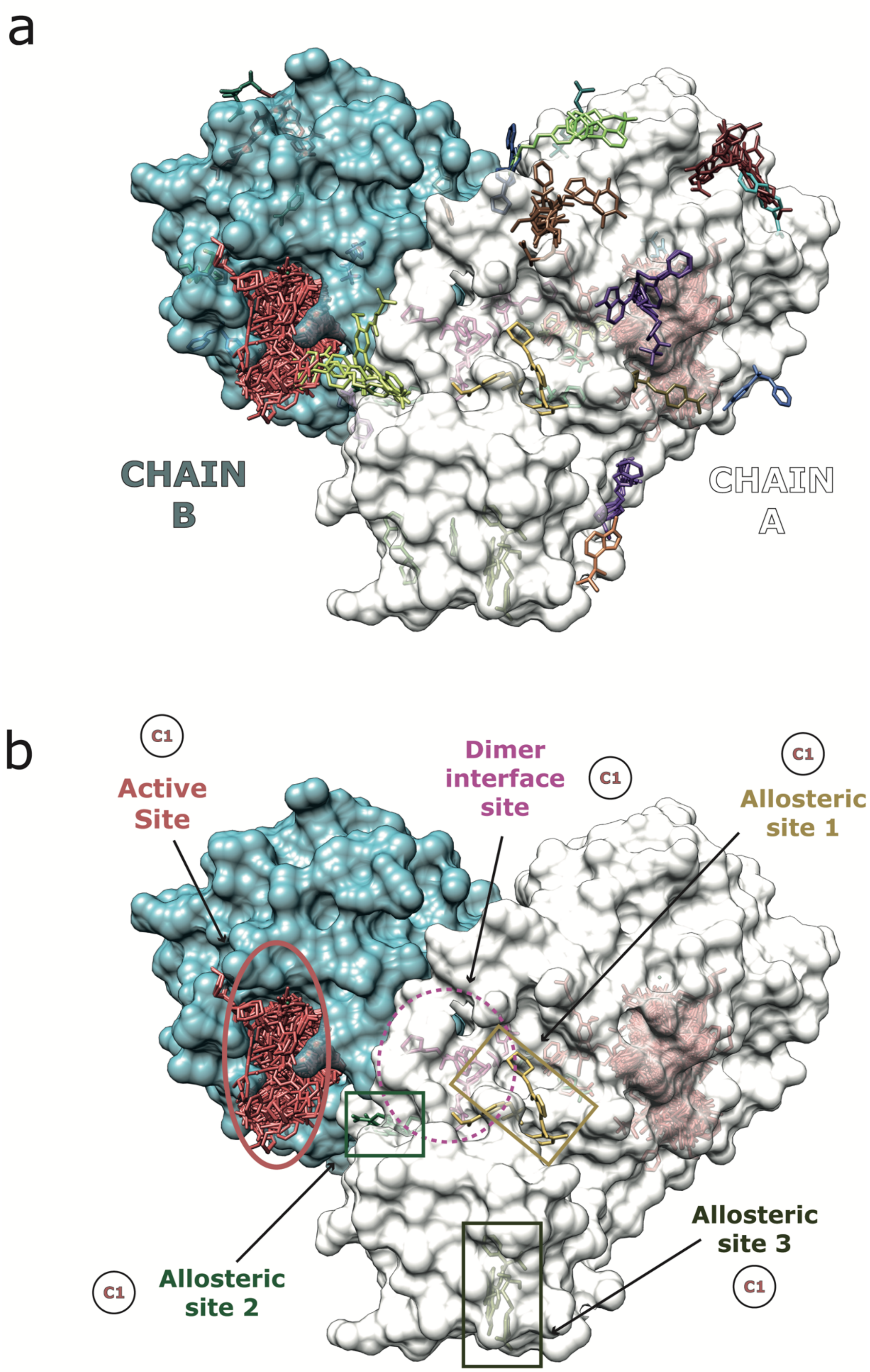

Supplementary Figure 4. (A) Twenty-five defined ligand binding sites on the SARS-CoV-2 main protease, MPro (P0DTD1) from 971 ligands from 511 structures; (B) Five of the 9 C1 sites included the known MPro active site, and four known potential allosteric sites [9, 10].

C1 site functional predictions supported by literature but not annotated in UniProt								
UniProt ID	RSA	$N_{Shenkin}$	MES	p	# residues	# ligands	UniProt residue numbers	Literature support
Q32ZE1	17.4	38.4	–0.21	0.02	10	1	[1762, 1763, 1765, 1766, 1769, 1791, 1991, 2034, 2035, 2038]	RNA binding [11] RNA exit site [12] D3 site [13]
Q9Y2J2	14.6	38.2	0.01	0.84	15	1	[117, 118, 119, 203, 206, 207, 210, 231, 232, 235, 236, 253, 282, 283, 286]	GPC binding [14]
Q9Y2J2	13.4	43.3	0.02	0.7	21	4	[154, 161, 162, 163, 164, 185, 186, 189, 208, 212, 217, 295, 297, 298, 299, 300, 301, 315, 375, 376, 379]	Calmodulin binding [14]
Q8WS26	16.2	28.9	–0.22	0.26	19	2	[105, 106, 107, 108, 109, 112, 151, 154, 155, 158, 159, 162, 170, 171, 173, 174, 175, 176, 179]	IPP, DMAPP binding [15, 16]
Q8WS26	22.1	31	0.18	0.58	8	2	[308, 312, 315, 316, 320, 324, 384, 423]	IPP binding [16]
P18031	20.8	33.9	0.05	0.48	14	1	[1, 2, 3, 4, 6, 10, 19, 242, 243, 244, 245, 246, 247, 271]	Conformational change [17] Cluster II [18]
P47811	17.1	55	0.08	0	19	10	[191, 192, 197, 198, 232, 236, 242, 246, 249, 250, 251, 252, 255, 259, 291, 292, 293, 294, 296]	MAP insert motif, Trp197 pocket [19, 20]

Q6B0I6	15.8	41.8	0.12	0.43	12	5	[193, 224, 225, 227, 228, 239, 240, 241, 242, 243, 277, 279]	Cryptic binding site [21]
P0DTD1	12.9	34.3	–0.13	0.45	12	2	[5501, 5503, 5809, 5810, 5811, 5838, 5839, 5840, 5841, 5856, 5858, 5878]	RNA binding [22]
P0DTD1	22.3	51.5	–0.04	0.87	9	1	[5806, 5809, 5810, 5811, 5839, 5874, 5876, 5878, 5879]	RNA binding [22]
P22557	16	47.8	–0.09	0.61	16	10	[148, 152, 155, 267, 268, 271, 272, 409, 413, 506, 570, 572, 573, 574, 575, 576]	Dimerisation interface [23]
P22557	12.7	53.1	0.08	0.61	7	2	[271, 293, 294, 295, 296, 297, 575]	Conformational change, PLP binding, succinyl-CoA inhibition [23]

Novel C1 cluster functional predictions

UniProt ID	RSA	$N_{Shenkin}$	MES	p	# residues	# ligands	UniProt residue numbers	Literature support
Q5T0W9	22.4	36.2	–0.24	0.08	12	10	[149, 150, 151, 177, 233, 234, 235, 236, 270, 273, 274, 277]	–
Q5T0W9	9.7	38.6	–0.05	0.79	12	2	[125, 126, 127, 129, 229, 255, 256, 257, 272, 275, 276, 279]	–
Q8WVM7	19.8	57.7	–0.23	0.62	5	1	[285, 288, 322, 325, 326]	–
Q15047	18.1	12.4	0.08	0.78	18	2	[295, 296, 297, 298, 300, 301, 302, 324, 328, 329, 330, 332, 333, 357, 389, 392, 393, 394]	–

Q8WS26	19.5	57.3	−0.11	0.57	21	26	[84, 87, 88, 89, 90, 214, 217, 218, 221, 222, 225, 268, 269, 273, 277, 281, 285, 290, 295, 299, 303]	–
Q9UGL1	28.7	31.3	−0.09	0.66	10	1	[53, 57, 506, 582, 583, 606, 607, 609, 610, 613]	–
Q9UGL1	16.6	34	−0.01	1	12	3	[658, 659, 662, 663, 666, 667, 670, 701, 736, 737, 738, 741]	–
P15379	18.3	19.4	0.09	0.63	11	1	[23, 24, 40, 41, 50, 146, 148, 162, 163, 164, 165]	–
Q9UJM8	24.3	42.8	−0.11	0.86	6	1	[5, 11, 323, 327, 328, 331]	–
Q6B0I6	21.9	36.6	−0.15	0.68	4	1	[50, 209, 265, 285]	–
Q6B0I6	12.2	26	−0.06	0.84	7	1	[44, 199, 275, 276, 297, 300, 303]	–
Q9UKK9	9.8	29.6	−0.05	0.73	15	1	[65, 66, 67, 69, 75, 77, 124, 125, 145, 146, 147, 175, 200, 205, 206]	–
Q92835	16.5	33.7	−0.05	0.78	19	46	[615, 616, 617, 618, 620, 621, 622, 624, 625, 630, 631, 632, 633, 634, 635, 636, 637, 638, 674]	–
Q92835	12.2	39.4	0.02	0.92	12	1	[560, 561, 562, 570, 571, 572, 573, 574, 578, 817, 839, 840]	–
Q96HY7	11.6	38.5	0.07	0.75	14	1	[57, 58, 60, 61, 64, 105, 106, 107, 121, 122, 125, 126, 147, 151]	–

P22557	17.5	40.6	0.04	0.72	16	7	[143, 145, 146, 149, 348, 349, 350, 351, 352, 353, 380, 381, 383, 402, 403, 406]	–
P24821	14.2	24.4	–0.29	0	15	8	[2010, 2011, 2012, 2025, 2045, 2046, 2047, 2048, 2049, 2050, 2054, 2055, 2056, 2057, 2060]	–

Supplementary Table 2. Twenty-nine RSA C1 ligand binding sites unannotated in UniProt, therefore classified as unknown function (UF). UniProt ID indicates the protein's UniProt accession. RSA is the median site RSA. $N_{SHENKIN}$ is the average normalised Shenkin score for the site. MES is the average missense enrichment score for the site. p is the p-value associated to this site MES. # residues is the number of residues forming the site. # ligands is the number of ligands binding to the site. UniProt residue numbers is a list of the UniProt residue numbers of the residues forming the site. Literature support contains a brief description of the site function and adequate references for the 12 sites (top) supported by the literature. The other 17 sites (bottom) represent novel predictions of functional sites.

Supplementary References

1. Ward, J.H., *Hierarchical Grouping to Optimize an Objective Function.* Journal of the American Statistical Association, 1963. **58**(301): p. 236-244.
2. Virtanen, P., et al., *SciPy 1.0: fundamental algorithms for scientific computing in Python.* Nat Methods, 2020. **17**(3): p. 261-272.
3. Lloyd, S., *Least squares quantization in PCM.* IEEE Transactions on Information Theory, 1982. **28**(2): p. 129-137.
4. Caliński, T. and J. Harabasz, *A dendrite method for cluster analysis.* Communications in Statistics, 1974. **3**(1): p. 1-27.
5. Davies, D.L. and D.W. Bouldin, *A Cluster Separation Measure.* IEEE Transactions on Pattern Analysis and Machine Intelligence, 1979. **PAMI-1**(2): p. 224-227.
6. Thorndike, R.L., *Who belongs in the family?* Psychometrika, 1953. **18**(4): p. 267-276.
7. Rousseeuw, P.J., *Silhouettes: A graphical aid to the interpretation and validation of cluster analysis.* Journal of Computational and Applied Mathematics, 1987. **20**: p. 53-65.
8. Benjamini, Y. and Y. Hochberg, *Controlling the False Discovery Rate: A Practical and Powerful Approach to Multiple Testing.* Journal of the Royal Statistical Society. Series B (Methodological), 1995. **57**(1): p. 289-300.
9. Douangamath, A., et al., *Crystallographic and electrophilic fragment screening of the SARS-CoV-2 main protease.* Nat Commun, 2020. **11**(1): p. 5047.
10. DasGupta, D., W.K.B. Chan, and H.A. Carlson, *Computational Identification of Possible Allosteric Sites and Modulators of the SARS-CoV-2 Main Protease.* J Chem Inf Model, 2022. **62**(3): p. 618-626.
11. Durgam, L. and L. Guruprasad, *Molecular mechanism of ATP and RNA binding to Zika virus NS3 helicase and identification of repurposed drugs using molecular dynamics simulations.* J Biomol Struct Dyn, 2022. **40**(23): p. 12642-12659.
12. Mottin, M., et al., *Molecular dynamics simulations of Zika virus NS3 helicase: Insights into RNA binding site activity.* Biochem Biophys Res Commun, 2017. **492**(4): p. 643-651.
13. Raubenolt, B.A., K. Wong, and S.W. Rick, *Molecular dynamics simulations of allosteric motions and competitive inhibition of the Zika virus helicase.* J Mol Graph Model, 2021. **108**: p. 108001.

14. Han, B.G., et al., *Protein 4.1R core domain structure and insights into regulation of cytoskeletal organization.* Nat Struct Biol, 2000. **7**(10): p. 871-5.
15. Munzker, L., et al., *Fragment-Based Discovery of Non-bisphosphonate Binders of Trypanosoma brucei Farnesyl Pyrophosphate Synthase.* Chembiochem, 2020. **21**(21): p. 3096-3111.
16. Gabelli, S.B., et al., *Structure and mechanism of the farnesyl diphosphate synthase from Trypanosoma cruzi: implications for drug design.* Proteins, 2006. **62**(1): p. 80-8.
17. Keedy, D.A., et al., *An expanded allosteric network in PTP1B by multitemperature crystallography, fragment screening, and covalent tethering.* Elife, 2018. **7**.
18. Cui, D.S., et al., *Leveraging Reciprocity to Identify and Characterize Unknown Allosteric Sites in Protein Tyrosine Phosphatases.* J Mol Biol, 2017. **429**(15): p. 2360-2372.
19. Francis, D.M., et al., *The differential regulation of p38alpha by the neuronal kinase interaction motif protein tyrosine phosphatases, a detailed molecular study.* Structure, 2013. **21**(9): p. 1612-23.
20. Nichols, C., et al., *Mining the PDB for Tractable Cases Where X-ray Crystallography Combined with Fragment Screens Can Be Used to Systematically Design Protein-Protein Inhibitors: Two Test Cases Illustrated by IL1beta-IL1R and p38alpha-TAB1 Complexes.* J Med Chem, 2020. **63**(14): p. 7559-7568.
21. Pearce, N.M., et al., *Partial-occupancy binders identified by the Pan-Dataset Density Analysis method offer new chemical opportunities and reveal cryptic binding sites.* Struct Dyn, 2017. **4**(3): p. 032104.
22. Newman, J.A., et al., *Structure, mechanism and crystallographic fragment screening of the SARS-CoV-2 NSP13 helicase.* Nat Commun, 2021. **12**(1): p. 4848.
23. Bailey, H.J., et al., *Human aminolevulinate synthase structure reveals a eukaryotic-specific autoinhibitory loop regulating substrate binding and product release.* Nat Commun, 2020. **11**(1): p. 2813.
